# The tale of caspase homologues and their evolutionary outlook: deciphering programmed cell death in cyanobacteria

**DOI:** 10.1093/jxb/eraa213

**Published:** 2020-05-05

**Authors:** Samujjal Bhattacharjee, Arun Kumar Mishra

**Affiliations:** 1 Laboratory of Microbial Genetics, Department of Botany, Banaras Hindu University, Varanasi, India; 2 University of Cambridge, India

**Keywords:** Altruistic adaptation, caspase homologues, horizontal gene transfer, orthocaspases, PCD morphotypes, programmed cell death

## Abstract

Programmed cell death (PCD), a genetically orchestrated mechanism of cellular demise, is paradoxically required to support life. As in lower eukaryotes and bacteria, PCD in cyanobacteria is poorly appreciated, despite recent biochemical and molecular evidence that supports its existence. Cyanobacterial PCD is an altruistic reaction to stressful conditions that significantly enhances genetic diversity and inclusive fitness of the population. Recent bioinformatic analysis has revealed an abundance of death-related proteases, i.e. orthocaspases (OCAs) and their mutated variants, in cyanobacteria, with the larger genomes of morphologically complex strains harbouring most of them. Sequence analysis has depicted crucial accessory domains along with the proteolytic p20-like sub-domain in OCAs, predicting their functional versatility. However, the cascades involved in sensing death signals, their transduction, and the downstream expression and activation of OCAs remain to be elucidated. Here, we provide a comprehensive description of the attempts to identify mechanisms of PCD and the existence and importance of OCAs based on *in silico* approaches. We also review the evolutionary and ecological significance of PCD in cyanobacteria. In the future, the analysis of cyanobacterial PCD will identify novel proteins that have varied functional roles in signalling cascades and also help in understanding the incipient mechanism of PCD morphotype(s) from where eukaryotic PCD might have originated.

## Teaser

A review of programmed cell death and its morphotypes in cyanobacteria, describing the functionality of orthocaspases based on distribution, domain architecture, mutations, and phylogeny, and their evolutionary and ecological significance.

## Introduction

The concept of cell death has been known to researchers for a long time from its physiological or pathological consequences. With the turn of century, the involvement of RNAs and proteins in cell demise mechanisms became clear as inhibition of transcription and translation was found to suppress cell death (Tata, 1966; Lockshin, 1969). On the other hand, development in microscopy techniques revealed a fixed set of morphological changes, such as blebbing, cell shrinkage, chromatin condensation (pyknosis), nuclear (karyorrhexis), and DNA fragmentation by dying cells, pointing towards a universally regulated process of cell death. Based on these physiological and biochemical attributes, Lockshin and Williams (1964) coined the term ‘programmed cell death’ (PCD). Kerr *et al.* (1972) recognized the biological pertinence of PCD and further classified it into two broad categories: apoptosis, an active and inherent programme of cell death induced by the cell itself or its surroundings; and necrosis, the passive and accidental collapse of the cell.

Today, the understanding of PCD in metazoans has reached a new height where the meaning of PCD has been broadened to subsume all the gene-dependent cell death mechanisms. This phenomenon of orchestrated cell demise is required by multicellular life forms for the sculpting of organs (such as interdigital regions in mammalian limbs; Jacobson *et al.*, 1997), removal of undesirable structures (as in the metamorphosis of tadpoles; Nishikawa and Hayashi, 1995; Jiang *et al.*, 1997), elimination of unhealthy or noxious cells (Cohen, 1991; Ju *et al.*, 1995; Asplund-Samuelsson *et al.*, 2012), and control of cell number, which pay off as enhanced overall fitness of the organism. Besides, the significance of PCD in stress and immune responses is also well recognized (Ameisen, 2002; Knight and Melino, 2011; Favaloro *et al.*, 2012; Green and Levine, 2014; Allocati *et al.*, 2015). As with animals, cell death is essential for plants as the process is involved in growth, differentiation, and stress responses. The terminal differentiation of tracheary elements, pod shattering, self-incompatibility-mediated pollen rejection, elimination of reproductive organs in unisexual flowers, and dehiscence of fruits are some examples (van Doorn and Woltering, 2005). Paradoxically, PCD has also been demonstrated in lower eukaryotes and several prokaryotes, suggesting a wider significance of this mechanism in maintaining life forms. Although the importance of PCD in multicellular organisms is evident, unfortunately understanding of PCD in the lower eukaryotes and prokaryotes is still limited, thereby to a large extent restricting our knowledge of the origin of this pre-eminent mechanism.

When PCD’s origin and emergence were considered, as its physiological importance was well-understood in multicellular systems, it was assumed to have originated with multicellularity. Subsequently, with the identification of diagnostic markers of early and late apoptosis in yeast (Madeo et al., 1997, 2002) and the finding of PCD in slime moulds (Cornillon *et al.*, 1994) and ciliates (Christensen *et al.*, 1995), it was concluded that actually multicellularity may not have been the most significant prerequisite for PCD, and later research revealed that unicellular organisms such as protozoa (Lee *et al.*, 2002), dinoflagellates (Vardi *et al.*, 1999), and bacteria (Lewis, 2000; Engelberg-Kulka, 2006; Rice and Bayles, 2008; Prozorov and Danilenko, 2011) also undergo PCD. The occurrence of bacterial PCD, characterized by a set of morphological and biochemical features, includes pyknosis, karyorrhexis, DNA fragmentation, proteolysis, and cell fragmentation (Hochman, 1997; Engelberg-Kulka *et al.*, 2006; Rice and Bayles, 2008), was postulated to be a protective mechanism against viral infections that later in evolution was acquired by unicellular eukaryotes and then multicellular systems (Ameisen, 2002; Dunin-Horkawicz *et al.*, 2014). Further studies showed that the death-specific factors in the bacteria are also induced by amino acid starvation (Schuster and Bertram, 2013), exposure to antibacterial compounds (Rice and Bayles, 2008), antagonism (Kim *et al.*, 2015), etc. Such reactions of microbial cells towards detrimental conditions indeed enhances the inclusive fitness through altruistic adaptation, i.e. by allowing a subset of cells to survive and reproduce at the expense of another subset (altruistic motifs) that is sacrificed during stress, thereby maintaining genetic diversity and fitness of the bacterial population (Durand *et al.*, 2016).

Like bacteria, cyanobacteria, a group of oxygenic photoautotrophs, are thought to display altruistic adaption through PCD. Unfortunately, cyanobacterial PCD has been affirmed by only a handful of experiments on *Anabaena* spp. (Ning *et al.*, 2002; Swapnil *et al.*, 2017), *Trichodesmium* spp. (Berman-Frank *et al.*, 2004), and *Microcystis aeruginosa* (Ding *et al.*, 2012; Klemenčič *et al.*, 2015), and thus the biochemical and molecular evidence to accompany the physiological observations is scanty. Therefore, we have taken an *in silico* approach along with wet-lab interpretations to describe PCD in cyanobacteria. This review seeks to identify the mechanisms of PCD, its morphotypes, and the death-responsive factors in cyanobacteria, and also compares it with PCD in metazoans and plants. Additionally, the importance of caspase homologues and their pseudo-variants in the cyanobacteria is discussed along with an analysis of the evolutionary and ecological dimensions of cyanobacterial PCD.

## History of programmed cell death research in cyanobacteria

**Fig. 1.**
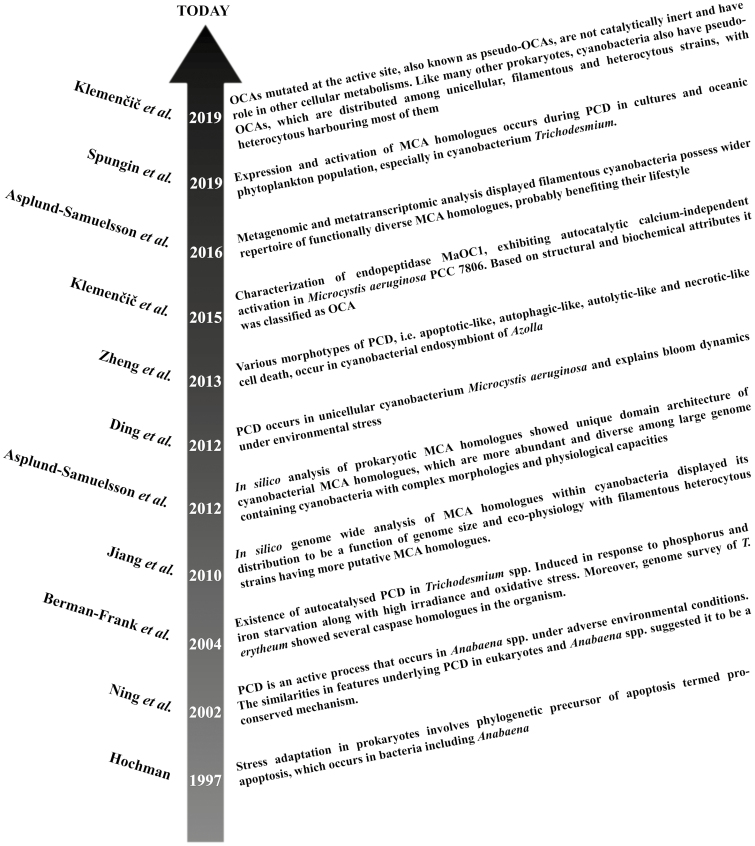
Timeline representation of major studies in the field of programmed cell death research in cyanobacteria and related organisms.

Proapoptosis is considered to be the phylogenetic precursor of apoptosis and is involved in population differentiation in prokaryotes, such as heterocyte formation in *Anabaena* and differentiation of *Rhizobium* into a bacteroid. Shortly after it was postulated by Hochman (1997), Ning *et al.*, (2002) showed various morphological and biochemical features of PCD in four species of *Anabaena*, i.e. *Anabaena* sp. PCC 7120, *A. cylindrica*, *A. siamensis*, and *A. flos-aquae*, upon salt stress. Soon afterwards, their equivalents were also identified in *Trichodesmium erythraeum* IMS101 under nutrient limitation, high irradiance, and oxidative stress (Berman-Frank *et al.*, 2004) and in *Microcystis aeuroginosa* upon hydrogen peroxide (H_2_O_2_) treatment (Ding *et al.*, 2012). Morphological and biochemical observations revealed the existence of PCD morphotypes, i.e. apoptotic-like, autophagic-like, autolytic-like, and necrosis-like cell death, in a cyanobacterial endosymbiont of *Azolla* at different developmental stages of the host plant (Zheng *et al.*, 2013). In parallel, caspase (cysteine-dependent aspartate-directed protease)-like activity, specific to metazoan apoptosis, was also detected in *T. erythraeum* IMS101 (Berman-Frank *et al.*, 2004) and *M. aeruginosa* (Ding *et al.*, 2012). The caspase homologues were classified as orthocaspases (OCAs) based on structural and functional differences observed during the characterization of MaOC1 in *M. aeruginosa* PCC 7806 (Klemenčič *et al.*, 2015). Besides, the direct correlation between the expression pattern and activity of caspase homologues with PCD was recently elucidated in *Trichodesmium* (Spungin *et al.*, 2019). A number of *in silico* approaches affirmed significant abundance of caspase homologues (mostly OCAs) in cyanobacteria (Asplund-Samuelsson *et al.*, 2012), with large-genome-containing, physiologically complex, heterocytous strains harbouring most of them (Jiang *et al.*, 2010; Asplund-Samuelsson *et al.*, 2012). Although OCAs have conserved histidine–cysteine (HC) dyads at the active site of a p20-like sub-domain that is a prerequisite for proteolysis, few of them have a mutated HC dyad and they are presumably involved in other forms of cellular metabolism (Asplund-Samuelsson, 2015; Klemenčič *et al.*, 2019). The timeline and progression of PCD research in the cyanobacteria is depicted in Fig. 1.

## Understanding programmed cell death in cyanobacteria

**Fig. 2.**
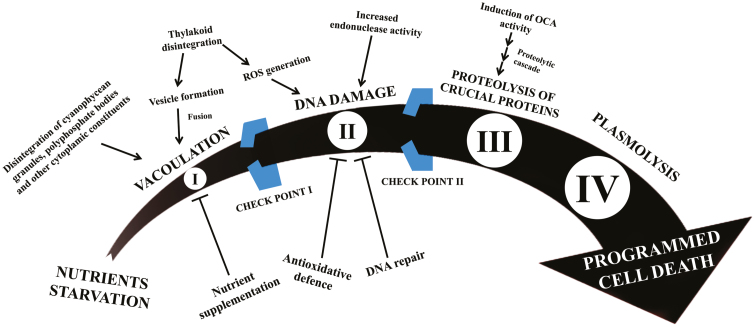
Progression of a cell through various characteristic events and two putative checkpoints (I and II) during programmed cell death (PCD) in cyanobacteria. Under nutrient starvation, vacuolation (stage I), due to disintegration of cellular components like cyanophycean granules, polyphosphate bodies, thylakoids, etc., initiates the mechanism of cell death; however, nutrient supplementation at an early stage (before passing through putative checkpoint I) may inhibit further progression into the death cascade. In the case of non-replenishment of nutrients, the DNA damage (stage II) in the cell would eventually occur as a consequence of intracellular reactive oxygen species formation due to various factors including thylakoid disintegration, although DNA repair mechanisms (before putative checkpoint II) can resist further progression of the cell towards death. Nevertheless, excessive DNA damage induces a putative wild-type orthocaspase (OCA) proteolytic cascade, identical to eukaryotic apoptosis, resulting in cleavage of crucial cellular proteins (stage III) including cytoskeletal proteins, metabolic enzymes, transcription factors, etc. Activation of an OCA proteolytic cascade leads to irreversible progression of the cell towards death largely due to the loss of important cellular proteins, which finally continues into PCD (stage IV). It is possible that under different environmental constraints initiation of PCD may vary, yet the basic scheme of programmed death should remain identical for cyanobacteria.

PCD in cyanobacteria is conferred by various morphological and biochemical equivalents of eukaryotic PCD in *Anabaena* spp. upon salt stress (Ning *et al.*, 2002). Salt induced disintegration of the plasmalemma and enhanced endonuclease activity resulted in DNA fragmentation, suggesting programmed death rather than lysis. Moreover, vacuolation in dying cells resulting from the fusion of several vesicles or disintegration of cellular constituents was also prominent. However, vacuolation could be reversed at an early stage by nutrient supplementation indicating the presence of various checkpoints in the mechanism of PCD, but once a cell passes through them, it would eventually die without any chance of reverting (Fig. 2). Although, vacuolation had already been reported in cyanobacteria upon ageing (Echlin, 1964; Lang and Rae, 1967; Peat and Whitton, 1967; Miller and Lang, 1971; Ermakova *et al.*, 1977), heat shock (Findley *et al.*, 1970), and heterocyte differentiation (Findley *et al.*, 1970; Kulasooriya *et al.*, 1972; Wilcox *et al.*, 1973; Roussard Jacquemin, 1983; Wolk *et al.*, 1994; Mishra *et al.*, 2018; Kaushik and Mishra, 2019), the exact reasons behind this are still unknown. Nevertheless, based on these observations the existence of a more complicated and conjoint mechanism leading to two opposite fates, cell death and heterocyte differentiation, can be speculated upon.

Several abiotic stresses induced PCD in various cyanobacterial species that was commonly characterized by vacuolation, thylakoid disintegration, disappearance of carboxysomes, gas vacuoles, and cyanophycean granules, cell shrinkage, elevated endonuclease activity, and DNA fragmentation. Phosphorus and nitrogen starvation along with high irradiance and oxidative stress activated PCD in *Trichodesmium* spp. (Berman-Frank *et al.*, 2004), while limited sulfur induced PCD in *Anabaena* sp. PCC 7120 (Kharwar and Mishra, 2020). Apoptotosis-like PCD was caused by exogenous H_2_O_2_ in *M. aeruginosa* (Ding *et al.*, 2012) and a salinity-induced oxidative burst in *A. fertilissima*, characterized by externalization of phosphatidylserine to the outer leaflet of the plasma membrane (Swapnil *et al.*, 2017). Additionally, in the dying cells of *T. erythraeum* IMS101 (Berman-Frank *et al.*, 2004) and *M. aeruginosa* (Ding *et al.*, 2012), caspase-like activity was determined by a caspase-specific substrate, DEVD (a peptide-based substrate that is equipped with a fluorophore and a cognate quencher so that caspase-mediated cleavage of DEVD produces fluorescence and the intensity can be correlated with active caspases in the cell). Interestingly, the caspase-like activity was not inhibited by z-VAD-FMK in *M. aeruginosa*, perhaps suggesting the presence of a caspase homologue lacking inhibitor sensitivity. However, with the rapid progress in knowledge of caspase homologues, caspase-specific substrates, like DEVD, should not be utilized to determine the activity of caspase homologues, since they have specificity for arginine (Arg) or lysine (Lys) instead of aspartate (Asp) at the P1 position. Recently, the caspase homologue MaOC1 in *M. aeruginosa* PCC 7806 was characterized as an orthocaspase that is activated by calcium-independent autocatalysis at Arg219, separating a 55 kDa catalytic subunit, and it displayed high catalytic efficacy for z-RR-AMC substrate (Klemenčič *et al.*, 2015).

## Comparing and recognizing various modes of cell death in cyanobacteria

In the early period of research, PCD was regarded as synonymous with apoptosis. However, this idea was confronted over the years with the recognition of other orchestrated cell death morphotypes that apparently displayed apoptosis as one PCD modality. The Nomenclature Committee on Cell Death (NCCD; Galluzzi et al., 2012, 2018) proposed a set of recommendations for functional classification of cell death subroutines and suggested molecular definitions of cell death modalities over morphological ones, since a high degree of biochemical, functional, and immunological heterogeneity may underlie similar morphological appearances. The classification defined cell death subroutines such as extrinsic apoptosis, intrinsic apoptosis, autophagic cell death, and mitotic catastrophe, along with a few additional cell death modalities (Galluzzi et al., 2012, 2018).

Extrinsic apoptosis is defined as a caspase-dependent process and it requires death receptor signalling, differentiating it from an intrinsic counterpart that depends on mitochondrial outer membrane permeabilization. Hence, intrinsic apoptosis is associated with dissipation of the mitochondrial transmembrane potential (∆ψ _m_) and release of proteins from the mitochondrial intermembrane space (Kroemer *et al.*, 2007). However, intrinsic apoptosis can be caspase-independent in the presence of caspase inhibitors, mediated by apoptosis-inducing factors and endonuclease G in an active manner or by ATP depletion in a passive manner (David *et al.*, 2006; Galluzzi *et al.*, 2012). Autophagic cell death, indicated by caspase-independent cell demise due to excessive autophagic flux and characterized by autophagosomes, is a general cytoprotective response that accelerates cell death (Kroemer *et al.*, 2009). Further, the idea of necrosis as an accidental death changed over the years, with the understanding of its regulation and significance in physiological and pathological responses (Vandenabeele *et al.*, 2010). Moreover, the term necroptosis has been recommended for use only for receptor-interacting protein kinase (RIP) 1- and 3-dependent necrosis (Galluzzi *et al.*, 2012). Recently, mitochondrial permeability transition-driven necrosis has been defined by disruption of ∆ψ _m_ due to loss of internal mitochondrial membrane impermeability and osmotic breakdown, leading to the necrotic morphotype (Galluzzi *et al.*, 2018). Additionally, mitotic catastrophe, considered as an onco-suppressive pathway, defines cell elimination during mitosis or subsequent interphase upon mitotic aberrations (Vakifahmetoglu *et al.*, 2008). Besides these, the following have also been described (Galluzzi et al., 2012, 2018): anoikis (loss of integrin-dependent attachment, inducing intrinsic apoptosis), autosis (autophagic cell death depending on the Na^+^/K^+^-ATPase on the plasma membrane), entosis (a cell-in-cell haemolytic interaction, degrading an engulfed cell within a lysosome), ferroptosis (cell death initiated by intracellular oxidative disturbances, inhibited by antioxidants or iron chelators), parthanatos (involving early activation of poly [ADP-ribose] polymerase 1), pyroptosis (with death dependent on caspase-1 and associated with a pyrogenic mediator like IL-1β and IL-18), NETosis (reactive oxygen species (ROS)-dependent cell death in neutrophils, associated with extrusion of neutrophil extracellular traps), and cornification (caspase-14-dependent death restricted to keratinocytes, involving lipid accumulation and extrusion).

In plants, PCD is known to be associated with developmental and defence mechanisms, yet detailed biochemical elucidation of the various PCD morphotypes that may occur, their classification, and their homology to animal system are arguable. Cell death during several developmental programmes in plants exhibited autophagic hallmarks (Beers, 1997), while protoplast retraction and DNA damage during other developmental, pathological, or stress responses are considered similar to apoptosis and termed apoptotic-like PCD (AL-PCD) (Danon,*et al.*, 2000). Though, caspase-like activity associated with the execution of AL-PCD (Reape and McCabe, 2008) indicated a close link with apoptosis, lack of any caspase-encoding genes and the presence of caspase homologues (three and six genes encoding metacaspase 1 and 2, respectively) in Arabidopsis (Watanabe and Lam, 2004) suggests a functionally similar yet non-identical mechanism of cell demise. However, the relocation of cytochrome *c* from mitochondria to the cytosol during cell death in stress conditions (Balk *et al.*, 1999) was not observed during AL-PCD (Balk *et al.*, 2003).

Recently, all the PCD classes known in plants have been broadly grouped into autolytic and non-autolytic cell death. However, apoptosis has been excluded, since plants lack phagocytes and the cell corpse does not disintegrate into apoptotic bodies (van Doorn, 2011). While the autolytic PCD is considered comparable to autophagic PCD in animals and characterized by rapid cytoplasmic clearance due to vacuolar hydrolase activity released during the process, the non-autolytic PCD, equivalent to necrosis, shows the absence of any such features, despite permeability enhancement and tonoplast rupturing in some cases. Generally autolytic PCD, related to developmental mechanisms, is predominant in senescent leaves, endosperm of germinating seeds, etc., where extensive degradation of complex biomolecules (DNA, RNA, proteins, carbohydrates) to simpler forms (sucrose, amides, amino acids) occurs. Besides, autolytic PCD responses include increased cytosolic Ca^2+^ concentration, nuclear condensation and fragmentation, DNA degradation, mitogen-activated protein kinase (MAPK) induction, along with enhanced caspase-like activity (van Doorn, 2011). Moreover, induction of the gene for cathepsin, a cysteine protease, is also evident during autolytic PCD in aleurone cells of barley (Martínez *et al.*, 2003). On the other hand, non-autolytic PCD underlies various defence responses as it is associated with vacuolar- and non-vacuolar-related hypersensitive responses and cell death due to necrotrophic pathogens; nonetheless, PCD in cereal endosperm takes a non-autolytic form (van Doorn, 2011). Vacuolar hypersensitive response-related PCD is often preceded by increased Ca^2+^ import, an oxidative burst, MAPK cascade induction and involvement of cysteine proteases (Belenghi *et al.*, 2003), whereas non-vacuolar hypersensitive response-related PCD in response to Victoria blight of oats is accompanied by caspase-like activity and reduced ∆ψ _m_ along with Ca^2+^ influx (van Doorn, 2011). Furthermore, in response to necrotrophic pathogens, increased intracellular H_2_O_2_ induced non-autolytic PCD (Williams and Dickman, 2008).

In spite of there being few morphological and biochemical similarities to the processes, the fundamental differences, such as absence of autolysosome- or autophagosome-like structures, apoptosome formation, or classical caspase genes, indicated a diverse origin and evolution of PCD in the two life forms. However, the ability of prime cell death proteases in plants (metacaspases) and animals (caspases) to cleave a phylogenetically conserved substrate, Tudor staphylococcal nuclease, argued for a common origin of PCD in plants and animals (Sundström *et al.*, 2009). Therefore, to understand the evolution of PCD in plants, the cell death morphotypes in cyanobacteria, the progenitors of oxygenic photoautotrophy, should be compared.

Studies in cyanobacteria have revealed various striking similarities with eukaryotic PCD, such as cytoplasmic clearance, phosphatidylserine externalization, DNA damage, and caspase-like activity. Nonetheless, these features are not distinguished into different PCD morphotypes, but rather subsumed into a common mechanism of cell death. However, a comprehensive study on the cyanobacterial endosymbiont of *Azolla microphylla* revealed the existence of four PCD morphotypes: apoptotic-like, autophagic-like, autolytic-like, and necrosis-like cell death (Zheng *et al.*, 2013). These PCD morphotypes have unique sets of ultrastructural and biochemical alterations, and hence can be easily distinguished. Externalization of phosphatidylserine detected by annexin V–green fluorescent protein-positive signals was evident only in apoptotic- and autophagic-like cell death, whereas DNA damage determined by a deoxynucleotidyl transferase dUTP nick end labelling assay is common to all dying cells. Furthermore, the detailed ultrastructural analysis through transmission electron microscopy effectively segregated the death morphotypes (Zheng *et al.*, 2013). The apoptotic-like cell death was characterized by cell shrinkage and thylakoid disintegration at an early stage but with intact outer membrane and peptidoglycan layer along with cellular inclusions such as cyanophycean granules and carboxysomes. These cells do not disintegrate into apoptotic bodies, but rather form a highly peculiar and irregular dendritic shape. The autophagic-like cell death was characterized by cytoplasmic disintegration and a high level of vacuolation. It was only during autolytic- and necrosis-like cell death that the peptidoglycan layer was lysed in the very early stage. While the former was characterized by cytoplasmic disintegration, generation of vesicles, and loss of cyanobacterial cytoskeleton (Guljamow *et al.*, 2012) in the late stages, the latter was characterized by rapid dispersal of cellular content upon cell wall rupturing and disruption of the actin meshwork (Table 1). These cell death morphotypes were common in all the cyanobacterial cell types, i.e. vegetative cells, heterocytes, and akinetes. Besides, apoptotic- and autophagic-like cell death predominantly occurred in the early developmental stage of the host plant, whereas the autolytic- and necrosis-like cell deaths were more evident at the later developmental stages of the fern.

**Table 1.** Distinguishing features of PCD morphotypes in cyanobacteria (adapted from Zheng *et al.*, 2013)

Cell death morphotypes		Apoptotic-like	Autophagic-like	Autolytic-like	Necrosis-like
Cyanobacterial cell type		Vegetative cells, heterocytes, akinetes	Vegetative cells, pro-heterocytes, heterocytes	Vegetative cells, heterocytes	Vegetative cells, heterocytes, akinetes
Cell morphology	Early response	Cell shrinkage commences	Swollen cell	Irregular shape, surrounded by a single membrane	Distorted cell
	Intermediate response	Irregular cell shape and reduced cell volume	Swollen cell	More irregularity in shape	Completely damaged cell
	Late response	Dendritic shape, reduced volume	Shape retained, empty cell	Shape lost, reminiscent of disintegrated cytoplasm	—
Cellular contents	Early response	Slight reduction	Slight reduction	Slight reduction	Cellular contents released or leaked out of the cell
	Intermediate response	More reduction	More reduction	Rapid reduction and loss of cellular contents	—
	Late response	Loss of cellular contents	Loss of cellular contents	Complete loss of cellular contents	—
Cell wall	Early response	Intact peptidoglycan	Intact outer membrane and peptidoglycan	Loss of peptidoglycan layer	Ruptured outer membrane, peptidoglycan remains intact, for akinetes and heterocyst outer envelope remains intact
	Intermediate response	Intact peptidoglycan	Intact outer membrane and peptidoglycan	Disintegration of cell wall components	Peptidoglycan disappearance
	Late response	Intact peptidoglycan	Intact outer membrane and peptidoglycan	Cell wall disappearance	—
Plasma membrane	Early response	Phosphatidylserine exposure, intact	Phosphatidylserine exposure, intact	Membranes begin to dissolve	Ruptured cell membrane
	Intermediate response	Invagination of membrane, intact	Onset of membrane rupture	Dissolution continues	—
	Late response	Irregular, intact	Complete rupture of cell membrane	Membrane vesiculation	—
Cytoskeleton		Disorganized pattern of actin	Not significant distortion of actin pattern	Loss of actin meshwork	Completely disrupted actin meshwork
Cytoplasm	Early response	Condensation commences	Condensation and vacuolation	Degradation commences	Leakage to extracellular space
	Intermediate response	Condensation continues	More cytoplasmic degradation and vacuolation	Rapid degradation	
	Late response	Maximum condensation	High level of vacuolation	Disintegration and leakage out	
Cytoplasmic inclusions (cyanophycean granules, carboxysomes)	Early response	Remain intact	No visible changes	Significant reduction of cellular inclusions	Loss of cellular inclusions
	Intermediate response	Remain intact	Dissolution of cytoplasmic inclusions	Significant reduction of cellular inclusions	
	Late response	Remain intact	Significant reduction of cellular inclusions	Loss of cellular inclusions	
Cell death morphotype markers		Intact cell wall and membrane while cell shrinkage and thylakoid membrane disintegrated at early stage	Cytoplasmic condensation and rapid vacuolation but cell wall remains intact	Disappearance of peptidoglycan layer in early stage and vesicles formed at later phage upon cytoplasm disintegration	Ruptured cell wall at early stage and cytoplasmic leakage
Cell death mechanism		Programmed	Programmed	Programmed	Not programmed

By a lenient comparison, there is a resemblance between the cyanobacterial autophagic- and necrosis-like cell death and autolytic and non-autolytic PCD in plants, yet apoptotic- and autolytic-like cell deaths have the least similarities to reconcile with any of the known PCD subroutines in plants. However, due to the lack of experimental evidence and mutually conserved comparable parameters because of fundamental differences between the life forms, the origin and evolution of PCD in phototrophs are still debatable. Therefore, studies on caspase homologues, prime executioners in these systems, are of value.

## Caspase homologues in cyanobacteria

Caspases are ubiquitous proteases, belonging to the peptidase C14 family of the CD clan, widely recognized to underpin apoptosis in the metazoans. The active caspases are dimeric proteins with each monomer having p20 catalytic and p10 regulatory sub-domains (Earnshaw *et al.*, 1999; Fuentes-Prior and Salvensen, 2004). The presence of an HC dyad within the p20 sub-domain confers cleavage specificity at the P1 Asp residue, and thus proteolytic activity. For a long time, the caspases were thought to be exclusive to metazoans, until the caspase-haemoglobinase-like fold, composed of four β-sheets surrounded by three α-helices (Aravind and Koonin, 2002), was also identified in non-metazoan caspase homologues (Uren *et al.*, 2000).

Caspase homologues, i.e. paracaspases (PCAs) (Jaworski and Thome, 2016) and metacaspases (MCAs) (Tsiatsiani *et al.*, 2011; Minina *et al.*, 2017), also harbour an evolutionarily conserved HC dyad within a p20-like sub-domain, a prerequisite for proteolytic activity, which divides the C14 family of proteases into two sub-families by the MEROPS database (https://www.ebi.ac.uk/merops/): C14A (caspases) (Shalini *et al.*, 2015) and C14B (MCAs and PCAs). However, the aspartate P1 cleavage specificity annotated for all the C14 members was lacking among these caspase homologues (McLuskey and Mottram, 2015). Therefore, this classification provided by the MEROPS database, based on the amino acid sequence similarities of the peptidase domain without considering their biochemistry (Rawlings *et al.*, 2018), has certain drawbacks (Minina *et al.*, 2020). Unlike caspases, PCAs are Arg specific and MCAs specifically cleave after either Arg or Lys residues. This fundamental difference among caspases, PCAs, and MCAs, contrary to the MEROPS descriptions, argues for the co-evolution of their substrates and pathways (Minina *et al.*, 2020). Furthermore, the active MCAs are monomeric calcium-dependent proteases (Hander *et al.*, 2019), whereas the PCAs, like caspases, are calcium-independent active dimers (Hachmann *et al.*, 2012), indicating differences in their activation and associated upstream mechanisms (Minina *et al.*, 2020). In consideration of this fundamental variability among caspases, MCAs, and PCAs, their recognition as three independent groups, with well-annotated structural and biochemical features, within the C14 family has recently been proposed (Minina *et al.*, 2020).

Moreover, domain configurations also displayed characteristic variabilities between MCAs and PCAs, and further divided them into three and two subgroups, respectively. PCAs lack a p10-like sub-domain but the prodomain is characterized by death and immunoglobin (Ig) domains (Uren *et al.*, 2000), whereas all three MCAs (type I, II, and III) harbour both p20- and p10-like sub-domains. The arrangement of sub-domains in type I and II MCAs is similar having p20- and p10-like sub-domains positioned at the N- and C-termini, respectively. Type I MCAs are characterized by proline-rich repeats and a zinc finger motif in the prodomain (Uren *et al.*, 2000) while type II MCAs are distinguished by an extended linker region (Vercammen *et al.*, 2004) and an additional linker within the p10-like sub-domain (Minina *et al.*, 2020). In contrast, type III MCAs, identified in algae and predicted to have emerged from secondary endosymbiosis, exhibited a rearrangement in the sub-domain architecture with a characteristic reverse orientation, i.e. p20- and p10-like sub-domains positioned at the C- and N-termini, respectively (Choi and Berges, 2013).

*In silico* studies of caspase homologues in bacterial phyla showed wide and scattered distribution, with an abundance among the members of the actinomycetes, α-, β-, and γ-proteobacteria, and cyanobacteria (Koonin and Aravind, 2002; Asplund-Samuelsson et al., 2012, 2016). However, these proteins are also found in Euryarchaeota, but at lower frequency. Generally, large-genome-containing bacteria harbour these proteases, yet a metagenomic study on their distribution in the Baltic Sea depicted that only 4% of the total bacterial population actually harbour them. Besides, caspase homologue genes were abundant among the filamentous cyanobacteria (Asplund-Samuelsson *et al.*, 2016). Genome-wide analysis of 33 cyanobacterial strains revealed the uneven distribution of caspase homologues (earlier termed MCAs), with morphologically and physiologically complex heterocytous strains having a larger share (Jiang *et al.*, 2010). Structural analysis of these cyanobacterial caspase homologues revealed the lack of a p10-like sub-domain (Choi and Berges, 2013). Additionally, characterization of a putative caspase homologue, MaOC1, from *M. aeruginosa* PCC 7806, showed it to exhibit calcium-independent autocatalytic activation after Arg219. Therefore, to distinguish them from other caspase homologues based on structural and functional attributes, they are termed orthocaspases (Klemenčič *et al.*, 2015). However, the detailed structure and biochemistry of OCAs has yet to be clarified (Minina *et al.*, 2020). Mostly the p20-like sub-domain-containing proteins are OCAs. MCAs were presumed to be absent in cyanobacteria (Choi and Berges, 2013) until recently when 50 sequences of type I MCAs were reported from a few species belonging to the genera *Anabaena*, *Calothrix*, *Coleofasiculus*, *Lyngbya*, *Moorea*, and *Nostoc*, accounting for merely ~4% of all cyanobacterial p20-like sub-domain-containing proteins (Klemenčič *et al.*, 2019). Among the cyanobacterial OCAs, 13% were found to be mutated at the functional HC dyad (Klemenčič *et al.*, 2019). Despite the paradigm suggesting these pseudo-variants (mutated OCAs; δOCAs) as catalytically inert due to a mutated active site, their distribution from unicellular (*Microcystis*) to filamentous (several genera of Nostocales) cyanobacteria (Klemenčič *et al.*, 2019) suggests their role in other cellular processes than cell execution. To gain insight into the role of wild-type OCAs and their pseudo-variants in cyanobacterial life, their evolutionary and ecological pertinence, a brief *in silico* analysis of their distribution, abundance, domain architecture, and phylogeny has been performed based on earlier predictions.

## *In silico* analysis

### Distribution and abundance of orthocaspases

**Fig. 3.**
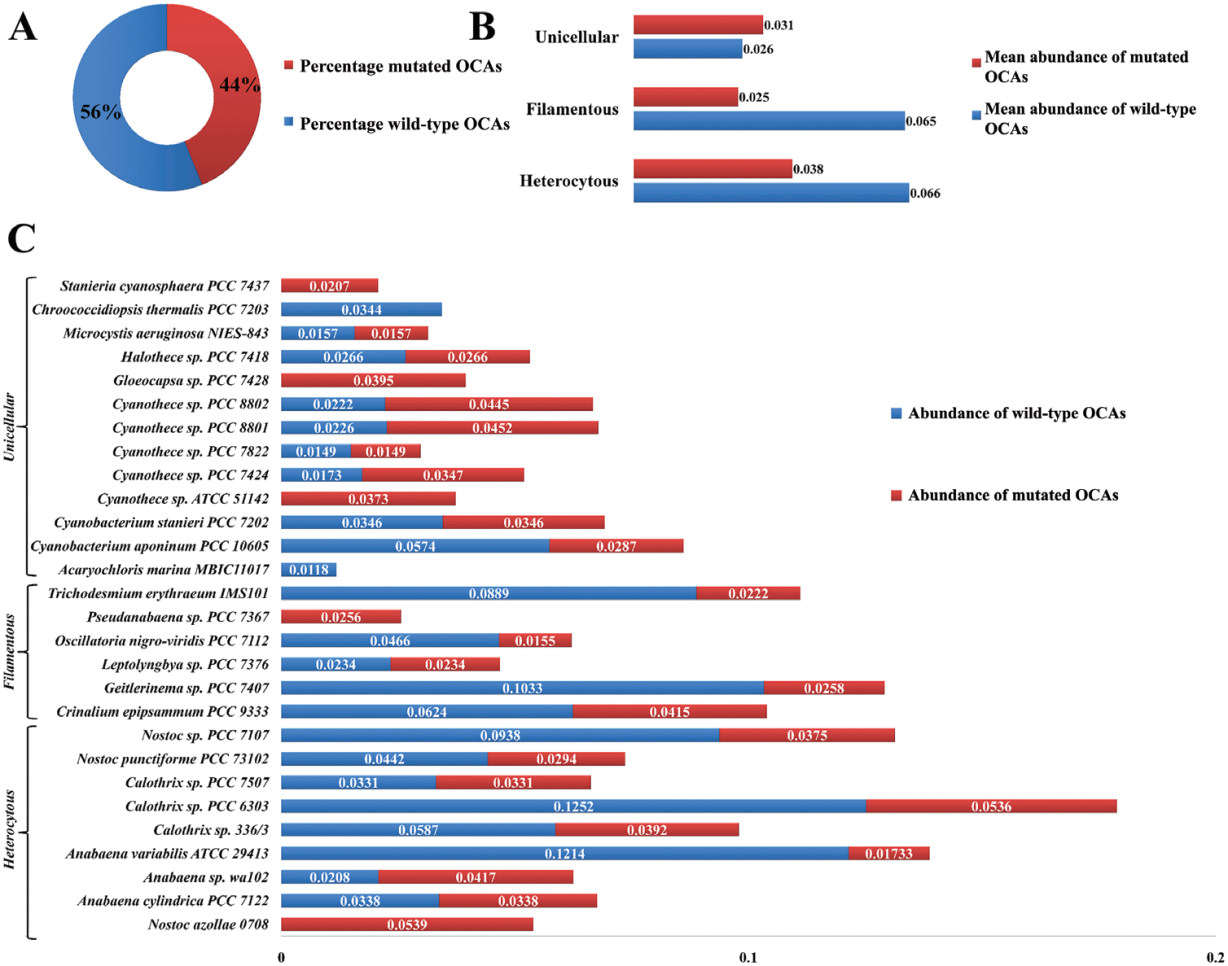
Distribution and abundance of orthocaspase subtypes among cyanobacteria (OCA per 100 proteins). (A) Percentage distribution of true OCAs and δOCAs among 29 analysed cyanobacterial strains showing that about 56% of all the OCAs are wild-type with conserved HC dyad and 44% are mutated at the active site. (B) Distribution and mean abundance of wild-type and mutated OCAs (δOCAs) among unicellular, filamentous, and heterocytous strains showing that the unicellular strains have lower and the filamentous and heterocytous strains have higher mean abundance of wild-type OCAs than their mutated variants. (C) The abundance of wild-type and mutated OCAs among 28 analysed cyanobacterial strains showing that morphological complexities, better physiological capacities, along with a larger genome favour the presence of wild-type OCAs, as heterocytous strains seemed to have more of them than other strains.

**Fig. 4.**
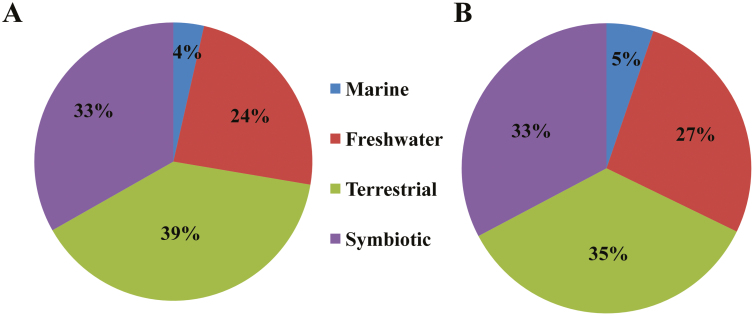
Habitat distribution of cyanobacterial strains harbouring wild-type orthocaspases (A) and mutated orthocaspases (δOCAs) (B).

Analysis of 85 cyanobacterial whole-genomes available in the CyanoBase (http://genome.microbedb.jp/cyanobase/) showed the distribution of 98 p20-like sub-domain-containing proteins (representing OCAs) among 33% of these strains (29 strains). Among these 98 OCAs, about 44% were pseudo-variants, having a mutated active site (HC dyad) (Fig. 3A). Further, 22 of these strains exhibited the presence of at least one copy of each wild-type and mutated OCA (OCA subtypes), whereas five strains, i.e. *Stanieria cyanosphaera* PCC7437, *Gloeocapsa* sp. PCC7428, *Cyanothece* sp. ATCC51142, and *Pseudoanabena* sp. PCC7367, harboured only δOCAs and two strains, i.e. *Acaryochloris marina* MBIC11017 and *Chroococcidiopsis thermalis* PCC7203, had wild-type OCAs only. Besides, the mean abundance of OCA was highest in heterocytous strains, having 0.066 OCA per 100 proteins, followed by filamentous and unicellular strains, with 0.065 and 0.026 OCA per 100 proteins, respectively. Interestingly, the mean abundance of δOCAs in the unicellular strains increased to 0.0317 per 100 proteins, whereas heterocytous and filamentous strains had 0.038 and 0.026 mean δOCAs per 100 proteins, respectively (Fig. 3B). It was also observed that the strains harbouring both OCA subtypes were abundant in freshwater and terrestrial habitats, whereas the marine habitat had only 4% and 5% of strains harbouring wild-type and mutated OCAs, respectively (Fig. 4A, B). The lack of OCAs from small-genome-containing marine strains of *Prochlorococcus*, *Synechococcus*, and *Synechocystis* corresponds to this observation.

The loss of OCA genes from the majority of these unicellular strains perhaps occurred during the course of evolution as these genes might not have conferred significant selective advantages to their lifestyle (Bidle and Falkowski, 2004). However, the putative OCAs in *Synechococcus* sp. JA-3-3Ab and *Synechococcus* sp. JA-2-3B′a(2–13), p20-like sub-domain-containing protein from *Synechococcus* sp. PCC 7942 (Jiang *et al.*, 2010), and a few δOCA encoding genes were reported from strains of *Synechococcus* and *Synechocystis* but no p20-like sub-domain-containing proteins were identified in highly streamlined picocyanobacteria, such as *Prochlorococcus* and *Cyanobium* (Klemenčič *et al.*, 2019). The absence of p20-like sub-domain-containing proteins from these picocyanobacteria might be a consequence of genome reduction for acclimatization to a sparse marine environment, which was previously reckoned for the loss of several DNA-repairing genes in *Prochlorococcus* spp. (Dufresne *et al.*, 2005). Besides, the terrestrial and freshwater habitats favoured the presence of OCAs (Jiang *et al.*, 2010). Moreover, the possibility of environmental extremes favouring the presence of at least one true OCA per genome has been observed in *Chroococcidiopsis thermalis* PCC 7203 and *Halothece* sp. PCC 7418, but no such proteases were reported in *Thermosynechococcus elongatus* BP-1 (Jiang *et al.*, 2010). Other selection pressures that favour the presence of OCAs include morphological and physiological complexities along with genome size, as larger-genome-containing heterocytous strains tended to harbour more OCAs (Jiang *et al.*, 2010). A closer look into the distribution of wild-type OCAs reflected that strains that displayed certain forms of sociability generally harbour them. Sociability can be described as systematic interactions among cells resulting in the formation of aggregates (*Cyanothece* sp.), colonies (*M. aeruginosa* NIES-843), or filaments, in which interdependency among individual cells resulted in a synchronized activity transforming them into a single unit. In such a situation the presence of true OCA activity seemed to induce altruistic adaptation whenever the population was subjected to harsh conditions.

Apart from free-living cyanobacteria, OCAs are also present in the symbiotic cyanobacterial strains *Nostoc azollae* 0708, *N. punctiforme* PCC 73102, and *A. marina* MBIC 11017. While *N. azollae* 0708 and *A. marina* MBIC 11017 have only mutated and wild-type OCAs, respectively, *N. punctiforme* PCC 73102 harbours both of them (Fig. 3C). Since in the symbiotic system the host generally regulates and maintains the symbiont population conferring homeostasis (Zheng *et al.*, 2013), the presence of wild-type OCAs in *N. punctiforme* PCC 73102 and *A. marina* MBIC 11017 may provide an additional way for the host to maintain the cyanobacterial population. Besides, these active proteases may induce PCD within the cyanobacterial symbiont under the highly stringent conditions inside the eukaryotic host (Zheng *et al.*, 2013), possibly providing another tier of adaptation through altruism. Further, it is also possible that PCD induced within the filament of *N. punctiforme* PCC 73102 would lead to the formation of hormogonia, the smallest unit of infection during establishment of symbiosis, in the presence of plant signals. On the other hand, the absence of any true OCA from *N. azollae* 0708 is possible because hormogonium formation is not required to establish the obligate symbiosis with *Azolla* sp. (Papaefthimiou *et al.*, 2008). Meanwhile, the δOCAs may confer other selective advantages yet to be revealed.

### Domain architecture among cyanobacterial orthocaspases

Cyanobacterial OCAs display variability in the whole protein domain architecture due to the presence of multiple domains in the same protein. Domain identification in these 98 OCAs using the Conserved Domain Database (https://www.ncbi.nlm.nih.gov/Structure/cdd/wrpsb.cgi) and PHMMER (https://www.ebi.ac.uk/Tools/hmmer/search/phmmer) exhibited 24 unique Pfam domains along with the p20-like sub-domain (peptidase C14) (see Supplementary Table S1 at *JXB* online) and a transmembrane domain. These additional domains associated with OCAs are involved in protein–protein interactions, protein modification, signalling, or PCD, or act as scaffold protein or chaperonins, whereas some domains have helicase and NTPase activity as well (Table 2), and thus provide OCAs with greater catalytic capacities and possibly confer their multifunctionality (Asplund-Samuelsson *et al.*, 2012). The basic structures of these OCAs are almost alike having the p20-like sub-domain positioned N-terminally with respect to accessory domains, except in Tery_1841 of *T. erythraeum*, which has the reverse orientation.

**Table 2.** Function of accessory domains of cyanobacterial orthocaspases

Serial no.	Additional domains	Description	Reference
1.	HEAT EZ	Scaffold protein associated with cyanobacterial phycobillisome lyase	Jiang *et al.* (2010)
2.	HEAT_2	Multiple HEAT repeats	
3.	WD40	Protein–protein interaction; involved in cell cycle control, apoptosis, and autophagy	Smith *et al.* (1999), Asplund-Samuelsson *et al.* (2012)
4.	eIF2A	Eukaryotic translation initiation factor 2A	
5.	TauB	ABC-type nitrate/sulfonate/bicarbonate transport system	
6.	AAA_16	Multifunctional protein having NTPase and chaperonin activity; involved in protein degradation, DNA replication, disassembly of proteins	Hanson and Whiteheart (2005), Tucker and Sallai (2007)
7	DUF	Domain of unknown function	
8	Abhydrolase	Contains catalytic triad of serine, glutamate/aspartate, histidine; displays serine protease-like activity	Remington *et al.* (1992)
9.	SAVED	SMODS-associated sensor domain; detects nucleotides and their derivatives	Burroughs *et al.* (2015)
10.	Sel1	Short repeats related to TRP; involved in protein–protein interactions	Asplund-Samuelsson *et al.* (2012)
11.	TRP	Tetratricopeptide repeats involved in protein–protein interactions	Blatch and Lässle (1999)
12.	PLN02742	Putative galactouronosyl transferase	
13.	Pentapeptide repeats	Sequence motifs found in multiple tandem repeats in protein molecules	Vetting *et al.* (2006)
14.	NACHT	Domain associated with PCD	Koonin and Aravind (2000)
15.	alas	Alanyl-tRNA synthetase-like domain	
16.	FGE-sulfatase	Formylglycine-generating sulfatase enzyme; involved in post-translational sulfatase modifications	Asplund-Samuelsson *et al.* (2012)
17.	MAP7	Domain associated with stabilization of microtubule protein	Fabre-Jonca *et al.* (1998)
18.	tolA	Inner membrane protein, required for cell envelope integrity	Lubkowski *et al.* (1999)
19.	Predic_Ig_Block	Putative immunoglobin-blocking virulence domain	
20.	PotA	Component of spermidine preferential uptake system, shows Mg^2+^ and SH-dependent ATPase activity	Kashiwagi *et al.* (1995)
21.	MIT_CorA like	Membrane protein involve in transportation of divalent cations across the membrane	
22.	PLN00181	SPA1-related domain	
23.	PRK06975	Bifunctional uroporphyrinogen-III synthetase domain	Guégan *et al.* (2003)
24.	GUN4	Intracellular signalling; also involved in accumulation of glycolipid in the heterocyte	Larkin *et al.* (2003), Jiang *et al.* (2010)
25	ANF-receptor	Transmembrane domain with extracellular ligand binding site	Jiang *et al.* (2010)
26	CHASE2	Transmembrane domain with extracellular sensor, involved in signal transduction	Jiang *et al.* (2010)
27.	DEXDc	Dead-like helicases involved in ATP-dependent DNA or RNA unwinding	Jiang *et al.* (2010)

**Fig. 5.**
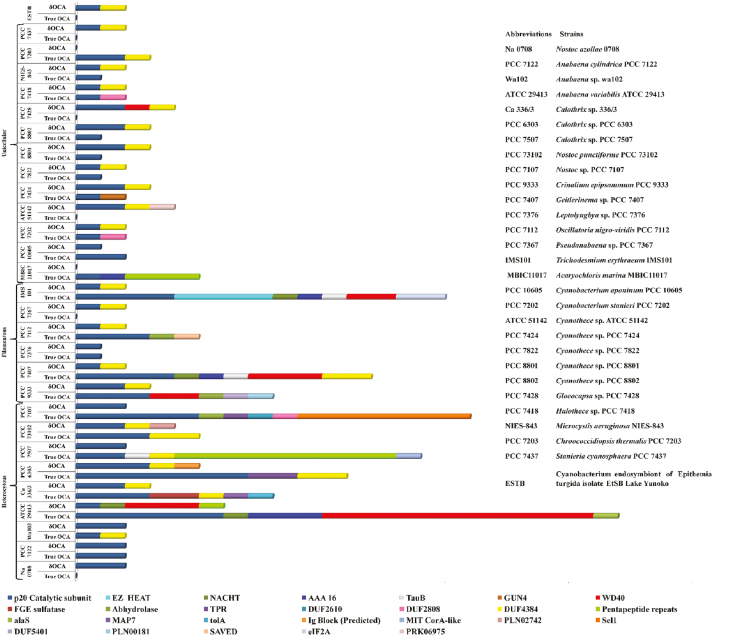
Distribution of p20-like sub-domain and accessory domains among wild-type and mutated orthocaspases in 29 analysed cyanobacterial strains (True OCA denotes wild-type orthocaspase and δOCAs denotes mutated orthocaspase). Domains are identified using the Conserved Domains Database of NCBI (https://www.ncbi.nlm.nih.gov/Structure/cdd/wrpsb.cgi) and PHMMER (https://www.ebi.ac.uk/Tools/hmmer/search/phmmer). It was observed that wild-type OCAs (true OCAs) have more domain variability than mutated OCAs (δOCAs). Moreover, the heterocytous strains also harbour greater domain variability than the other strains.

Among the cyanobacterial OCA subtypes, the wild-type OCAs had wider variability of accessory domains compared with their mutated variants. Most of the δOCAs had only a few identifiable accessory domains such as domain of unknown function (DUF4384) and WD40, except Ava_2183 and Npun_F0844, which had NACHT and PLN02742 domains, respectively (Fig. 5). The distribution of accessory domains was also uneven among cyanobacteria, since the OCAs from heterocytous strains with broader physiological capacities exhibited the presence of diverse accessory domains and architectures (Fig. 5).

Considering the occurrence of different domain types among OCAs, but disregarding successive repeats in a single protein, three domains, i.e. WD40 (9%), AAA_16 (6%), and NACHT (4%), appeared most. Additionally, DUF4384, DUF2808, DUF2610, and pentapeptide repeats were also associated with several OCAs (Fig. 5). Among all the 24 domains, 11 domains consisting of GUN4, alas, MAP7, tolA, PotA, Ig block (predicted), SAVED, MIT CorA-like, PLN02742, PLN00181, and PRK06975 were particularly rare as each of them was associated with one OCA only. Domains like CHASE2 (Jiang *et al.*, 2010; Asplund-Samuelsson *et al.*, 2012), ANF-receptor (Jiang *et al.*, 2010; Asplund-Samuelsson *et al.*, 2012), and DEXDc (Jiang *et al.*, 2010), which were previously reported in some cyanobacterial OCAs, were not identified in these 98 sequences. Transmembrane domains were also present in 25 OCAs among which Tery2624, Osc7112_3570, and PCC7424_0759 have the transmembrane domain positioned N-terminally to the p20-like sub-domain. Thus, it can be inferred that the OCAs can either be cytoplasmic (lacking a transmembrane domain) or membrane-bounded (containing a transmembrane domain).

The prevalence of WD40 in most of the OCAs suggested their interaction with the target proteins may be carried out via this domain. The presence of NACHT in Tery_1841, Ava_2184, and GEI7407_1529 indicated their putative roles in PCD or related pathways. Interestingly, the co-occurrence of NACHT and WD40 domains in the wild-type OCAs, i.e. Ava_2184 and GEI 7407_1529, except Tery_1841, may indicate protein–protein interactions during proteolytic cleavage. OCAs containing the wild-type p20-like sub-domain and WD40 at N- and C-termini, respectively, flanking a transmembrane domain, probably have an extracellular localization of WD40 leaving the p20-like sub-domain free for catalysis in the cytoplasm. This extracellular WD40 may interact with the specific growth or death regulatory proteins, and probably modulates the activity of the p20-like sub-domain in the predicted cytoplasmic portion. This presumed topology prevailed in six true OCAs: Ava_0039, Ava_3079, Cri9333_3253, GEI7407_1667, GEI7407_1667, and Glo7428_1095. Among these, the presence of NACHT in the predicted cytoplasmic portion of GEI7407_1667 implies its activity during PCD. Moreover, Ava_0039, Ava_3079, and GEI7407_1667 have an AAA_16 domain placed N-terminally to the transmembrane domain, probably indicating energy-dependent activity of OCAs. Nevertheless, three putative cytoplasmic OCAs consisting Tery_2471, AMI_G0162, and Ava_2184 also have an AAA_16 domain providing NTPase and chaperone activities. The prevalence of abhydrolase, a domain having serine protease-like activity due to the catalytic triad of Ser, Glu/Asp, and His in Osc7112_0662, Cri9333_0714, and Nos7107_1322 inclusive of the p20-like sub-domain probably implies dual specificity of OCA (Spungin *et al.*, 2019), ensuring a wider range of target proteins. While the first two have a transmembrane domain and are predicted to be membrane-bound, the latter may have a cytoplasmic localization. Further, the presence of formylglycine-generating enzyme sulfatase and GUN4 may confer on the respective OCAs post-translational sulfate modifying and signalling attributes. The features of these accessory domains that have been analysed in these 98 OCAs or previously reported in cyanobacterial OCAs are shown in Table 2.

**Fig. 6.**
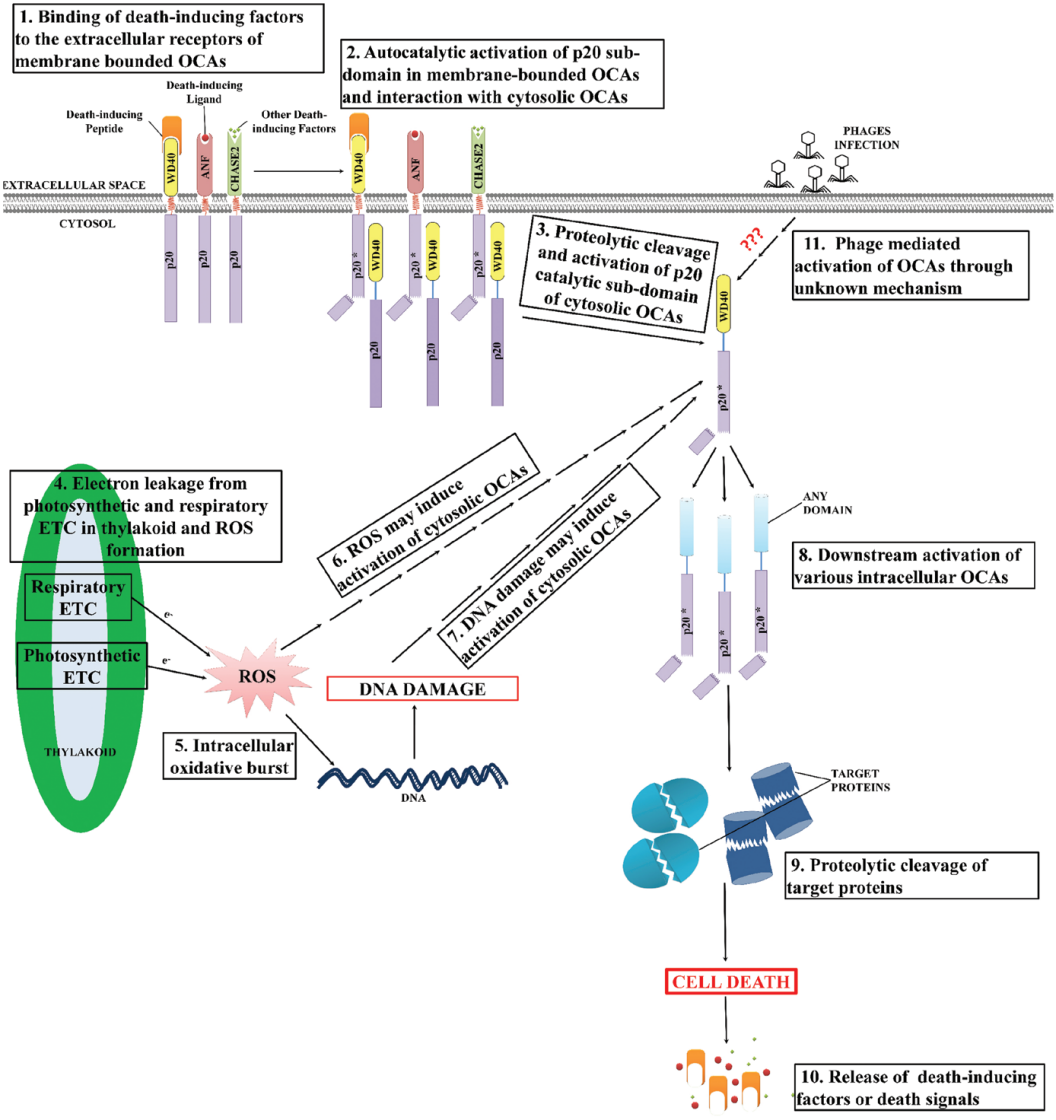
Putative mechanism of orthocaspase (OCA)-mediated programmed cell death (PCD) in cyanobacteria (here, OCAs denote only the wild-type OCAs involve in the proteolytic cascade). (1) Death-inducing peptides, ligands, and other factors bind to WD40, ANF, and CHASE2 domains, respectively, at the surface of target cells. These domains are a part of the membrane-bound OCAs having cytosolic localization of p20-like sub-domains. (2) Binding of these factors to the receptors induces autocatalysis and activation of the p20-like sub-domain, (3) resulting in downstream activation of cytosolic OCAs by proteolysis. The interaction of membrane-bound p20-like sub-domain with cytosolic OCAs may occur via the WD40 domain of the latter. However, the activation of cytosolic OCAs may be due to (4, 5, 6) ROS generation resulting in an oxidative burst and (7) subsequent DNA damage. Such activation of cytosolic OCAs either by extracellular or by intracellular signals leads to (8) downstream activation of a proteolytic cascasde and (9) cleavage of target proteins ultimately leading to cell death. Further, (10) several death-inducing factors can be released by the cell upon death. (11) It is also possible that cyanophages also induce a similar proteolytic cascade leading to PCD during phage infection in cyanobacteria. To avoid confusion created by diverse types of accessory domain in the OCA, due to their diverse functionalities and scope to participate in an array of cellular process, only ANF receptor, CHASE2, and WD40 along with the p20-like catalytic sub-domain have been shown, and all other domains are non-specifically represented by ‘Any Domain’.

The occurrence of predicted cytoplasmic and membrane-bound wild-type OCAs may suggest the existence of both intrinsic and extrinsic PCD in cyanobacteria equivalent to metazoan apoptosis (Fig. 6). Although, these mechanisms are less appreciated in prokaryotes, the domain topology of several OCAs predicts their propagation. It is possible that the cyanobacteria harbouring both transmembrane and cytosolic wild-type OCAs, such as *T. erythraeum* IMS101, *Oscillatoria nigro-viridis* PCC 7112, and *Nostoc* sp. PCC 7107, may induce PCD either by intracellular (DNA damage, oxidative burst) or by external (nutrient limitation, high temperature, irradiance) factors, whereas PCD in the cyanobacteria having only cytosolic wild-type OCAs, such as *C. stanieri* PCC 7202 and *C. thermalis* PCC 7203, depends only on an intracellular stimulus. Further, the presence of extracellular domains may result in neighbouring cells being engaged in PCD as well (Asplund-Samuelsson *et al.*, 2012). Further, the cytoplasmic and transmembrane wild-type OCAs probably act equivalent to executioner and initiator caspases, respectively, in a putative proteolytic cascade. Possibly, upon external stimulus or a death signal received by the extracellular domain of a transmembrane OCA, the cytosolic wild-type p20-like sub-domain undergoes autocatalysis to activate itself and simultaneously activates downstream cytosolic wild-type OCAs, thereby inducing a proteolytic cascade (Fig. 6). Nevertheless, experimental evidence is still required to ascertain the existence of such a mechanism in cyanobacteria.

### Mutation and phylogeny

**Fig. 7.**
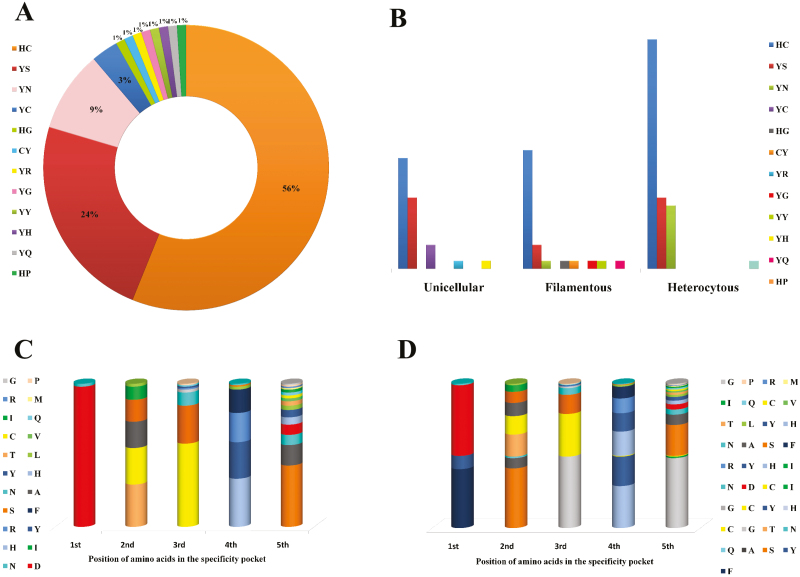
Mutations at specificity pockets and active sites of orthocaspses (OCAs). (A) Percentage occurrence HC dyad and its mutated variants at the active site of OCAs in 98 OCA sub-types. (B) Distribution of HC dyad and its mutated variants among unicellular, filamentous, and heterocytous strains. (C, D) Mutations at of H-specificity pocket (C) and C-specificity pocket (D).

Alignment of these 98 p20-like sub-domains displayed a high degree of variability within the sequences (Supplementary Figs S1, S2; Bidle and Falkowski, 2004). The HC dyad was found to be conserved in 56%, whereas 44% had a mutated catalytic site, corresponding to wild-type and mutated OCAs, respectively (Fig. 7A). Substitution of H by Y (tyrosine) at an H-catalytic site was observed in 40 sequences, with one sequence having C in place of H. On the other hand, C was replaced by S (serine) in 26 sequences, while N (asparagine) and G (glycine) substituted C in nine and two sequences, respectively, at the C-catalytic site. Moreover, R (arginine), Y, Q (glutamine), and P (proline) also substituted C in one sequence each (Supplementary Figs S1, S2). Therefore, the substitution of the HC dyad by YS and YN is more common than substitution with YR, YY, YQ, HP, HG, YC, CY, YG, and YH dyads (Fig. 7A). Furthermore, substitution of HC by FS (phenylalanine–serine) and YA (tyrosine–alanine) was previously observed in rare cyanobacterial δOCAs (Klemenčič *et al.*, 2019). Additionally, more variability at the active site was observed for filamentous strains, followed by unicellular ones whereas heterocytous strains had lesser variability (Fig. 7B). Mutations not only replaced the HC dyad but also other residues in the ‘specificity pocket’ that is formed by the amino acids flanking these specific H and C residues and determine substrate specificity (Uren *et al.*, 2000). Although, a significant order of mutation was observed in the specificity pockets (Fig. 7C, D), the consensus sequence was found to be FSG(H/Y)G and D(T/C/A/S)(C/S)(H/Y/R)(S/N) for the H- and C-pockets, respectively. Further, a higher order of substitution at C- than H-specificity pockets was also observed. The loss of Arg- and Lys-specific proteolytic activity (Jiang *et al.*, 2010) and their probable role in PCD due to mutation may not necessarily erode their role in cellular metabolism (Asplund-Samuelsson, 2015) as depicted by pseudopeptidase MCA4 (with C substituted by S) of *Trypanosoma brucei*, crucial for virulence and cell cycle progression (Proto *et al.*, 2011), and further indicates the multi-functionality of these pseudo-proteases. However, the δOCAs in cyanobacteria are presumed to be catalytically inert and therefore their role in other cellular metabolism has escaped attention.

**Fig. 8.**
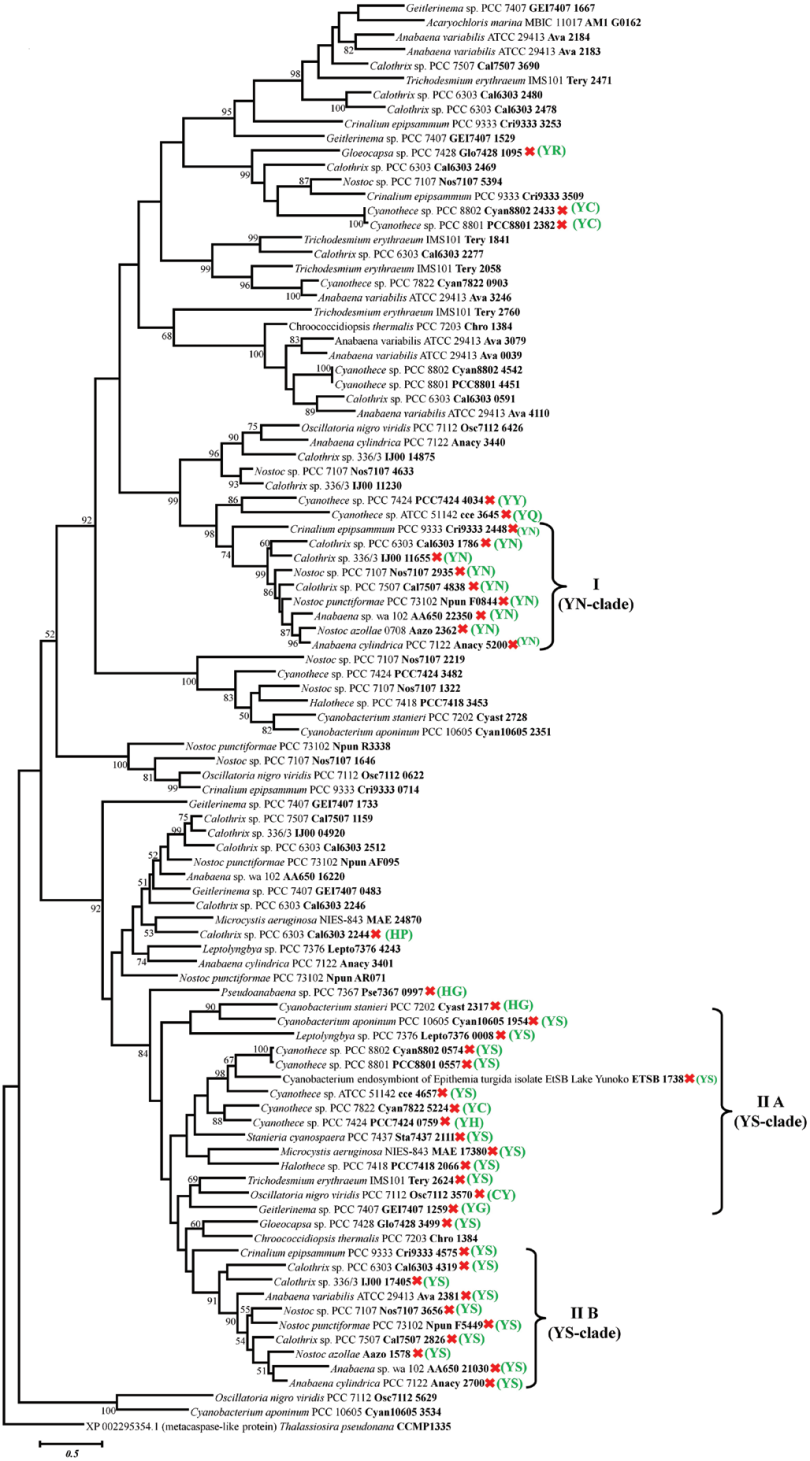
Phylogenetic relationship of 98 p20-like catalytic sub-domains of OCAs obtained from 29 cyanobacterial strains. The phylogenetic tree was constructed using the maximum likelihood method of MEGA 7 with metacaspase-like protein from *Thalassiosira pseudomana* CCMP1335 as outgroup. Bootstrap values of more than 50 are indicated (1000 replicates). Cyanobacterial strains and protein IDs are shown in the tree. Mutated p20-like sub-domains are represented by a cross and mutated active site amino acid dyads are shaded. Two clades representing mutated p20-like sub-domains are shown, i.e. the YN clade (I) and YS clade (II), with the latter divided into two sub-clusters, IIA and IIB. While clade I and IIB all have YN and YS dyads at mutated active sites, IIA have a majority of YS dyads with few variants. Other forms of mutated active sites were distributed along the tree. Moreover, cluster I and sub-cluster IIB mostly have heterocytous strains, except *Crinalium epipsammum* PCC 9333, whereas sub-cluster IIA has heterocytous, filamentous, and unicellular strains.

Phylogenetic analysis of these p20-like sub-domains displayed intermixing of unicellular, filamentous, and heterocytous strains (Fig. 8). Therefore, the p20-like sub-domain phylogeny is incongruent with the 16S gene-based phylogeny and perhaps points towards horizontal gene transfer (HGT), which may have led to the acquisition of these proteases in the cyanobacteria, as predicted earlier (Bidle and Falkowski, 2004). Besides, the distant positioning of several p20-like sub-domains of a single strain, such as Ava_2184, Ava_3246, and Ava_3079 from *A. variabilis* ATCC 2943 or Tery_2471, Tery_1841, and Tery_2760 from *T. erythraeum* IMS101, is apparently explained by multiple independent HGT events during the course of evolution. However, the tree displayed two distinct δOCA clusters denoting YN (I) and YS (II) clades (Fig. 8). Further, the YS clade can be sub-divided into two sub-clusters: IIA and IIB. While IIA consisted majorly of YS-substituted p20 sequences along with one p20 sequence each for HG, YH, YC, CY, and YG substitutions, IIB represented a pure YS-substituted p20-like sub-domain sub-cluster (Fig. 7). Interestingly, both cluster YN (I) and sub-cluster YS (IIB) contained mainly heterocytous strains, except *Crinalium epipsammum* PCC 9333 representing the only non-heterocytous strain in both the clusters, whereas sub-cluster YS (IIA) was composed of heterocytous, filamentous, and unicellular δOCAs. Apart from that, all other p20 variants from different strains are scattered throughout the tree. Such a phylogenetic arrangement of δOCA has not only conferred functional variability but may also indicate a diverse origin and independent emergence. This probably implies the acquisition of OCA subtypes in the cyanobacteria from multiple donors harbouring different OCAs. It is also possible that the mutation has occurred in cyanobacteria after HGT to acquire adaptive advantages during evolution. Yet no conclusion can be drawn without explicit studies in the context that has crafted the present scenario.

## Evolutionary advantages of programmed cell death in cyanobacteria

Although, the acquisitions of OCAs in the cyanobacterial genome have occurred through putative HGT, their stable incorporation and maintenance in the host genome argues for their evolutionary and adaptive significance. The prime impediment to recognizing the significance of these genes in cyanobacteria is the maintenance of suicide-responsive genes in isolated cells offering no benefits. However, to understand the explicit impact of PCD, a broader scenario beyond the cell should be considered. In microbial communities, PCD is defined as an adaptive mechanism to death induced by environmental constraints that alter the microenvironment, affecting the interactions among various taxa (Durand *et al.*, 2016; Durand and Ramsey, 2019). The impact of PCD on microbial communities was evident in the *Dunaliella–Halobacterium* model (Orellana *et al.*, 2013). It was shown that the organic substances released during PCD in *Dunaliella salina* were reutilized subsequently by living cells and re-mineralized by *Halobacterium salinarum*. PCD and consequential benefits of nutrient exchange were also shared by bacterial phytoplankton communities, where organic ingredients released by phytoplankton during PCD were utilized by bacteria (Azam and Malfatti, 2007). Nevertheless, the *Chlamydomonas* model revealed that the benefits of PCD were obvious for individuals from the same species (Durand *et al.*, 2014). The cellular substances released upon PCD in *Chlamydomonas reinhardtii* CC125 enhanced fitness of *C. reinhardtii* UTEX89 but significantly inhibited *Chlamydomonas moewusii* and *Chlamydomonas debaryana*, also suggesting a role of PCD in inter-specific competition. In bacteria, the coupling of the toxin–antitoxin system with PCD modules during stress suggested a correlation of PCD with bacterial immunity (Yamaguchi *et al.*, 2011; Koonin and Zhang, 2017). Additionally, PCD in bacterial populations offers differentiation, development of multicellular structures, and enhanced durability of colonies under unfavourable conditions (Dewachter *et al.*, 2015). Therefore PCD, a negative selection for individual cells, confers adaptive advantages on the population (Pepper *et al.*, 2013).

In cyanobacteria, the idea of adaptive advantages conferred by PCD is rudimentary, urging explicit research; however, stress-induced PCD has suggested its vital role in adaptation. Under environmental constraints PCD may act to relieve the stressed population by eliminating aged or damaged cells, thereby releasing nutrients for the younger cells to utilize (Kohanski *et al.*, 2010). Though PCD is the final choice once the energy expenditure for damage repair exceeds the cost of forming new cells in the population (Bayles, 2014), it has evolved as a strategy ensuring population stability. Such altruistic behaviour in a cyanobacterial population allows the survival of a subset of the population at the expense of others under unfavourable conditions, thereby enhancing inclusive fitness.

PCD in prokaryotes was initially thought to be a viral defence strategy. Protection against the T4 phage in *E. coli* is conferred by killing of the host before the completion of the T4 lytic cycle, which commences with the detection of Gol T4 peptide in the host leading to activation of the death cascade (Georgiou *et al.*, 1998). This altruistic behaviour resists the propagation of the phage infection and enables survival of the population at the minimal cost of a few cells. Further, *E. coli* populations with the PCD genotype have a greater survival and fitness than those without when subjected to phage infection (Refardt *et al.*, 2013). Similarly, phage infection can possibly be resisted through the onset of PCD in and around infected cells in cyanobacteria. Death of host cells upon attachment or penetration would subsequently restrict viral transcription and biosynthesis of phage particles upon loss of the host transcription machinery, enhancing fitness of the cyanobacterial population against lytic phages.

The role of PCD in propagule formation is largely unrecognized, since no studies explicitly discuss the process, but a few observations presumably denote such instances. The collapse of the *Peridinium gatunense* bloom is a seasonal phenomenon occurring due to changes in environmental cues that induce PCD and, subsequently, release thiol esterase (a signalling peptide) that further induces rapid cell death (Vardii *et al*., 2007). This supports the survival of a few individual cells in the stressful conditions, and these are assumed to serve as propagules for the next blooming season. In snowflake yeast, the death of interior cells causes segregation of the parent colony into daughter colonies that can grow into new parent colonies (Ratcliff et al., 2012, 2015). In filamentous cyanobacteria, such as *Trichodesmium*, *Oscillatoria*, and *Nostoc*, the formation of hormogonia (small groups of vegetative cells released from the parent filament, serving both as germinating propagules and infective units to establish plant–cyanobacterial symbiosis) may be considered as an outcome of sequential PCD in the parent filament (Bidle and Falkowski, 2004), induced not only by desiccation, cold stress, or phosphate starvation, but also in the presence of plant signals, although precise experimental proof is still required.

## Ecological significance of cyanobacterial programmed cell death

Cyanobacteria are a major component of the global carbon and nitrogen cycles and therefore their PCD would certainly influence both the energetic and the biogeochemical cycles, which is sorely overlooked (Franklin *et al.*, 2006). Cyanobacteria transfer energy not only through the predation pathway in marine ecosystems but also to conspecific microbes upon PCD. PCD in the *Trichodesmium* bloom causes release of transparent polysaccharides and a downward flux of organic nutrients. Such a phenomenon enables efficient supply of nutrients, especially carbon and nitrogen, to the deep sea (Bar-Zeev *et al.*, 2013). Thus, PCD in cyanobacteria facilitates flow and flux of organic resources in the microbial loop through an unconventional non-predation pathway (Scavia and Fahnenstiel, 1987; Franklin *et al.*, 2006; Bidle, 2016), and therefore can probably be considered to have been associated with an ecologically significant incipient mechanism of energy transfer in the primitive ocean before the existence of predators.

## Conclusion and future perspective

Iterative bioinformatics and experimental analysis support the existence of a stringent biochemical and molecular core underlying cyanobacterial PCD. Despite fundamental differences, the presence of OCAs and several eukaryotic PCD equivalents suggests a similar mechanism of cell demise in cyanobacteria. Moreover, the evolutionary significance of PCD in providing another tier of adaptation to the cyanobacterial population through altruism, along with its ecological pertinence, has also been perceived. Besides, the crucial involvement of δOCAs in various cellular mechanisms other than cell execution has also been speculated upon. However, the field is young and questions regarding death cascades, executioners, and related factors are still to be addressed. Therefore, explicit research and thoughtful experimentation will pave the way to elucidating factors involved in and mediating this intriguing and imperative process in cyanobacteria. Possibly with this, the origin and evolution of PCD in phototrophs will be deciphered.

## Supplementary data

Supplementary data are available at JXB online.

Fig. S1. Multiple sequence alignment of ‘histidine (H)-specificity pocket’ of 98 p20-like catalytic sub-domains of OCAs obtained from 29 cyanobacterial strains.

Fig. S2. Multiple sequence alignment of ‘cysteine (C)-specificity pocket’ of 98 p20-like catalytic sub-domains of OCAs obtained from 29 cyanobacterial strains.

Table S1. Different domains of cyanobacterial orthocaspases (OCAs) and their positions.

eraa213_suppl_Supplementary_Table_S1_Figures_S1-S2

## Abbreviations

δOCA: mutated orthocaspase
HC: histidine–cysteine
HGT: horizontal gene transfer
MCA: metacaspase
OCA: orthocaspase
PCA: paracaspase.
