# Supplementary material for: The tale of caspase homologues and their evolutionary outlook: deciphering programmed cell death in cyanobacteria
Source: J Exp Bot. 2020 May 5;71(16):4639–57. doi: 10.1093/jxb/eraa213 (PMC7475262; doi:10.1093/jxb/eraa213)
Supplement: eraa213_suppl_Supplementary_Table_S1_Figures_S1-S2 [file eraa213_suppl_supplementary_table_s1_figures_s1-s2.pdf]

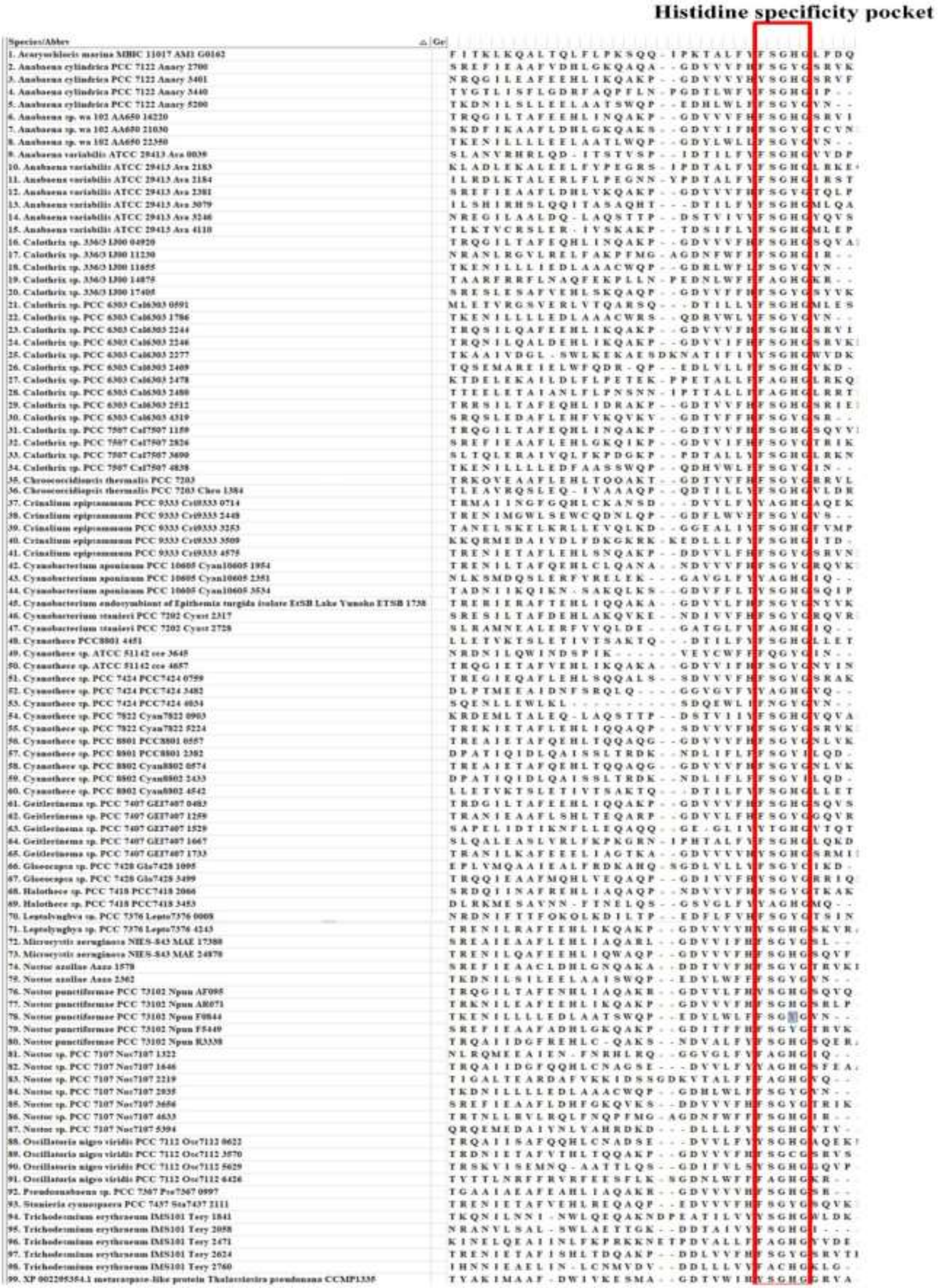

Supplementary Fig. S1. Multiple sequence alignment of "Histidine (H)-specificity pocket" of 98 p20 catalytic sub-domains of OCAs obtained from 29 cyanobacterial strains.

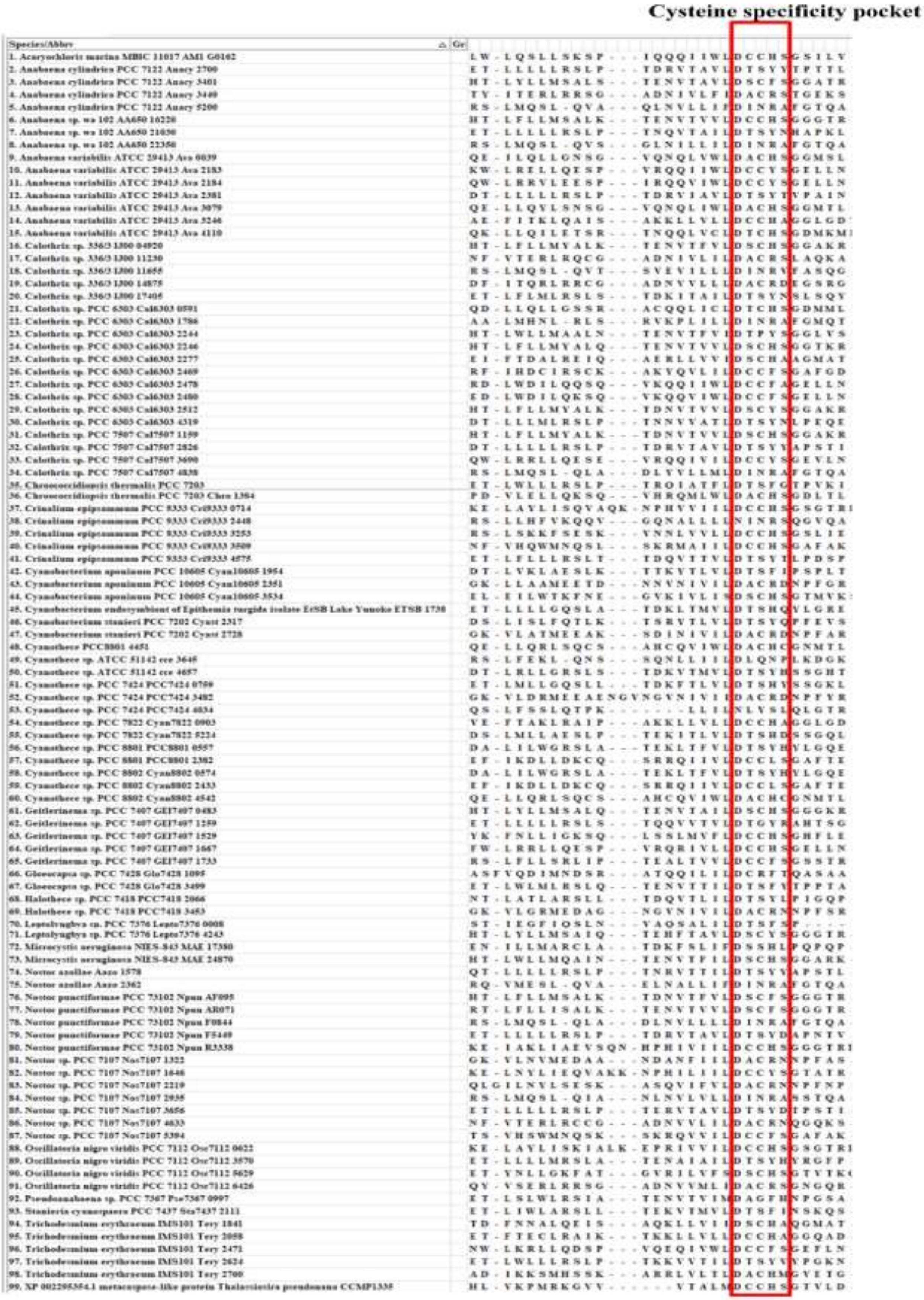

Supplementary Fig. S2. Multiple sequence alignment of "Cysteine (C)-specificity pocket" of 98 p20 catalytic sub-domains of OCAs obtained from 29 cyanobacterial strains.

Table S1. Different domains of cyanobacterial orthocaspases (OCAs) and their positions.

S.No.	Cyanobacterial strains	Orthocaspases (OCAs)	Pfam Domains		
			From	To	Short name
1	*Trichodesmium erythraeum* IMS101	Tery_1841	581	910	HEAT EZ
			7	421	NACHT
			511	596	HEAT_2
			883	968	
			476	534	
			1026	1282	Peptidase C14
		Tery_2058	8	236	Peptidase C14
		Tery_2471	809	1087	WD40
			1099	1375	
			891	1006	eIF2A
			1006	1126	
			7	288	Peptidase C14
			287	332	TauB
			295	436	AAA_16
		Tery_2624 [p]	44	279	Peptidase C14

			628	727	DUF4384
		Tery_2760	144	383	Peptidase C14
2	*Stanieria cyanospaera* PCC 7437	Sta7437_2111 [p]	50	312	Peptidase C14
			600	686	DUF4384
3	*Pseudoanabaena* sp. PCC 7367	Pse7367_0997 [p]	622	705	DUF4384
			51	151	Peptidase C14
4	*Oscillatoria nigro viridis* PCC 7112	Osc7112_0622	6	292	Peptidase C14
			977	1022	Abhydrolase
		Osc7112_3570 [p]	44	298	Peptidase C14
			565	650	DUF4384
		Osc7112_5629	4	254	Peptidase C14
		Osc7112_6426	4	241	Peptidase C14
			379	570	SAVED
5	*Nostoc* sp. PCC 7107	Nos7107_1322	359	500	DUF2808
			51	272	Peptidase C14
		Nos7107_1646	5	272	Peptidase C14
			856	991	Abhydrolase
		Nos7107_2219	701	778	DUF2610
			56	287	Peptidase C14
			337	579	TPR
			344	453	Sel1 (3 repeats, 2 repeats, 2 repeats
			459	536	

			582	649	)
		Nos7107_2935 [p]	4	228	Peptidase C14
		Nos7107_3656 [p]	42	291	Peptidase C14
		Nos7107_4633	4	240	Peptidase C14
		Nos7107_5394	3	197	Peptidase C14
6	*Nostoc punctiformae* PCC 73102	Npun_AF095	595	679	DUF4384
			45	318	Peptidase C14
		Npun_AR071	45	273	Peptidase C14
			581	657	DUF4384
		Npun_F0844 [p]	4	227	Peptidase C14
			354	527	PLN02742
		Npun_F5449 [p]	42	290	Peptidase C14
			543	635	DUF4384
		Npun_R3338	6	283	Peptidase C14
7	*Nostoc azollae* 0708	Aazo_1578 [p]	45	292	Peptidase C14
		Aazo_2362 [p]	4	224	Peptidase C14
8	*Acaryochloris marina* MBIC 11017	AM1_G0162	830	869	Pentapeptide repeats (3 repeats)
			906	943	
			945	982	
			5	235	Peptidase C14
			282	403	AAA_16
9	*Anabaena cylindrica* PCC 7122	Anacy_2700 [p]	42	292	Peptidase C14

		Anacy_3401	45	201	Peptidase C14
		Anacy_3440	4	243	Peptidase C14
		Anacy_5200 [p]	4	234	Peptidase C14
10	*Anabaena* sp. wa 102	AA650_16220	595	678	DUF4384
			45	316	Peptidase C14
		AA650_21030 [p]	42	145	Peptidase C14
		AA650_22350 [p]	4	216	Peptidase C14
11	*Anabaena variabilis* ATCC 29413	Ava_0039	1067	1353	WD40
			1234	1603	
			1606	1643	
			1358	1394	
			1397	1434	
			23	242	Peptidase C14
			528	665	AAA_16
		Ava_2183 [p]	1104	1388	WD40
			941	1220	
			1272	1525	
			444	787	NACHT
			5	186	Peptidase C14
			900	936	Pentapeptide repeats
		Ava_2184	821	1105	WD40

			1115	1146	
			5	239	Peptidase C14
			652	894	NACHT
			280	427	AAA_16
		Ava_2381	45	296	Peptidase C14
		Ava_3079	1278	1601	WD40
			1492	1734	
			1137	1396	
			1445	1472	
			22	254	Peptidase C14
			550	732	AAA_16
			1058	1209	alaS
		Ava_3246	8	236	Peptidase C14
		Ava_4110	23	287	Peptidase C14
12	*Calothrix* sp. 336/3	IJ00_04920	584	668	DUF4384
			42	289	Peptidase C14
		IJ00_11230	401	653	FGE-sulfatase
			4	240	Peptidase C14
		IJ00_11655 [p]	4	214	Peptidase C14
		IJ00_14875	515	761	FGE-sulfatase
			4	240	Peptidase C14
			324	367	MAP7

			294	391	tolA
		IJ00_17405 [p]	42	258	Peptidase C14
			557	648	DUF4384
13	*Calothrix* sp. PCC 6303	Cal6303_0591	23	287	Peptidase C14
		Cal6303_1786 [p]	4	214	Peptidase C14
			414	571	predic_Ig_block
		Cal6303_2244 [p]	45	323	Peptidase C14
		Cal6303_2246	45	274	Peptidase C14
			589	675	DUF4384
		Cal6303_2277	11	256	Peptidase C14
		Cal6303_2469	3	231	Peptidase C14
		Cal6303_2478	5	156	Peptidase C14
		Cal6303_2480	308	349	TRP (2 repeats)
			449	490	
			3	254	Peptidase C14
		Cal6303_2512	591	674	DUF4384
			42	305	Peptidase C14
		Cal6303_4319 [p]	42	286	Peptidase C14
			540	628	DUF4384
14	*Calothrix* sp. PCC 7507	Cal7507_1159	584	668	DUF4384
			42	307	Peptidase C14
		Cal7507_2826 [p]	49	300	Peptidase C14

		Cal7507_3690	790	1067	Pentapeptide repeats
			767	804	
			5	239	Peptidase C14
			325	439	PotA
			275	429	TauB
			567	688	MIT_CorA-like
		Cal7507_4838 [p]	4	228	Peptidase C14
15	*Chroococcidiopsis thermalis* PCC 7203	Chro_0010	42	328	Peptidase C14
			611	700	DUF4384
		Chro_1384	41	283	Peptidase C14
16	*Crinalium epipsammum* PCC 9333	Cri9333_0714	6	298	Peptidase C14
			908	1021	Abhydrolase
		Cri9333_2448 [p]	4	241	Peptidase C14
		Cri9333_3253	783	1074	WD40
			956	1378	
			3	244	Peptidase C14
			1088	1291	PLN00181
		Cri9333_3509	3	241	Peptidase C14
			279	507	DUF5401
		Cri9333_4575 [p]	59	332	Peptidase C14
			645	721	DUF4384
17	*Cyanobacterium aponinum* PCC 10605	Cyan10605_1954 [p]	42	150	Peptidase C14

		Cyan10605_2351	74	296	Peptidase C14
		Cyan10605_3534	4	270	Peptidase C14
18	Cyanobacterium endosymbiont of Epithemia turgida isolate	ETSB_1738	45	275	Peptidase C14
			595	680	DUF4384
19	*Cyanobacterium stanieri* PCC 7202	Cyast_2317 [p]	47	130	Peptidase C14
			564	639	DUF4384
		Cyast_2728	336	477	DUF2808
			61	282	Peptidase C14
20	*Cyanothece* sp. ATCC 51142	cce_3645 [p]	7	197	Peptidase C14
			273	435	PRK06975
		cce_4657 [p]	45	305	Peptidase C14
			576	661	DUF4384
21	*Cyanothece* sp. PCC 7424	PCC7424_0759 [p]	53	327	Peptidase C14
			622	715	DUF4384
		PCC7424_3482	337	476	GUN4
			56	285	Peptidase C14
		PCC7424_4034 [p]	4	153	Peptidase C14
22	*Cyanothece* sp. PCC 7822	Cyan7822_0903	8	236	Peptidase C14
		Cyan7822_5224 [p]	53	323	Peptidase C14
			646	737	DUF4384
23	*Cyanothece* sp. PCC 8801	PCC8801_0557 [p]	45	254	Peptidase C14
			560	638	DUF4384

		PCC8801_2382 [p]	3	240	Peptidase C14
		PCC8801_4451	23	261	Peptidase C14
24	*Cyanothece* sp. PCC 8802	Cyan8802_0574 [p]	45	254	Peptidase C14
			560	638	DUF4384
		Cyan8802_2433 [p]	3	242	Peptidase C14
		Cyan8802_4542	27	261	Peptidase C14
25	*Geitlerinema* sp. PCC 7407	GEI7407_0483	569	654	DUF4384
			42	324	Peptidase C14
		GEI7407_1259 [p]	623	717	DUF4384
			45	135	Peptidase C14
		GEI7407_1529	773	1102	WD40
			3	227	Peptidase C14
			267	314	TauB
			293	401	NACHT
		GEI7407_1667	1269	1561	WD40
			882	1149	
			5	225	Peptidase C14
			270	417	AAA_16
		GEI7407_1733	42	196	Peptidase C14
			593	661	DUF4384
26	*Gloeocapsa* sp. PCC 7428	Glo7428_1095 [p]	324	605	WD40
			3	200	Peptidase C14

		Glo7428_3499 [p]	42	320	Peptidase C14
			589	682	DUF4384
27	*Halothece* sp. PCC 7418	PCC7418_2066 [p]	51	314	Peptidase C14
			589	679	DUF4384
		PCC7418_3453	451	592	DUF2808
			41	261	Peptidase C14
28	*Leptolyngbya* sp. PCC 7376	Lepto7376_0008 [p]	44	143	Peptidase C14
		Lepto7376_4243	14	283	Peptidase C14
29	*Microcystis aeruginosa* NIES-843	MAE_17380 [p]	40	277	Peptidase C14
			526	616	DUF4384
		MAE_24870	45	207	Peptidase C14

Domains are identified using PHMMER (https://www.ebi.ac.uk/Tools/hmmer/search/phmmer)and Conserve domain database of NCBI (https://www.ncbi.nlm.nih.gov/Structure/cdd/wrpsb.cgi)

[p] denotes mutated-orthocaspase
